# GTP binding controls complex formation by the human ROCO protein MASL1

**DOI:** 10.1111/febs.12593

**Published:** 2013-11-28

**Authors:** Sybille Dihanich, Laura Civiero, Claudia Manzoni, Adamantios Mamais, Rina Bandopadhyay, Elisa Greggio, Patrick A Lewis

**Affiliations:** 1 Department of Molecular Neuroscience, UCL Institute of NeurologyLondon, UK; 2 Department of Biology, University of PadovaItaly; 3 Reta Lila Weston Institute and Queen Square Brain Bank, UCL Institute of NeurologyLondon, UK; 4 School of Pharmacy, University of ReadingUK

**Keywords:** complex formation, G-protein, HSP60, LRRK2, MASL1, ROCO

## Abstract

The human ROCO proteins are a family of multi-domain proteins sharing a conserved ROC-COR supra-domain. The family has four members: leucine-rich repeat kinase 1 (LRRK1), leucine-rich repeat kinase 2 (LRRK2), death-associated protein kinase 1 (DAPK1) and malignant fibrous histiocytoma amplified sequences with leucine-rich tandem repeats 1 (MASL1). Previous studies of LRRK1/2 and DAPK1 have shown that the ROC (Ras of complex proteins) domain can bind and hydrolyse GTP, but the cellular consequences of this activity are still unclear. Here, the first biochemical characterization of MASL1 and the impact of GTP binding on MASL1 complex formation are reported. The results demonstrate that MASL1, similar to other ROCO proteins, can bind guanosine nucleotides via its ROC domain. Furthermore, MASL1 exists in two distinct cellular complexes associated with heat shock protein 60, and the formation of a low molecular weight pool of MASL1 is modulated by GTP binding. Finally, loss of GTP enhances MASL1 toxicity in cells. Taken together, these data point to a central role for the ROC/GTPase domain of MASL1 in the regulation of its cellular function.

**Structured digital abstract:**

- HSP60 and MASL1 physically interact by molecular sieving (View interaction)
- MASL1 physically interacts with HSP70 and HSP60 by anti tag coimmunoprecipitation (View interaction)
- MASL1 physically interacts with HSP60 by anti tag coimmunoprecipitation (View interaction)

## Introduction

**Figure 1:**
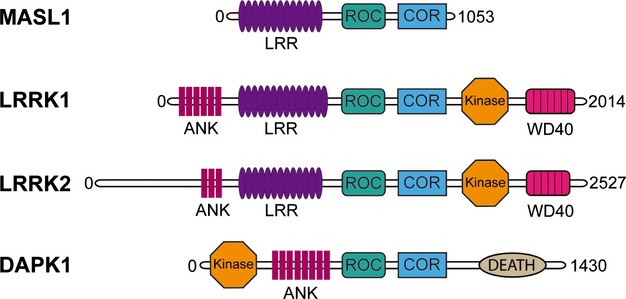
The human ROCO protein family consists of four proteins called LRRK1, LRRK2, DAPK1 and MASL1. Together a conserved GTPase domain called ROC-COR, which stands for Ras of complex proteins and C-terminal of ROC domains, characterizes these proteins.

Malignant fibrous histiocytoma amplified sequences with leucine-rich tandem repeats 1 (MASL1) is the smallest member of the human ROCO protein family, members of which are characterized by a conserved supra-domain containing a Ras of complex proteins (ROC) domain and a C-terminal of ROC (COR) domain [1]. In addition to MASL1, this protein family includes the leucine-rich repeat kinases 1 and 2 (LRRK1/2) and death-associated protein kinase 1 (DAPK1) (Fig. 1) [2]. All four of the human ROCO proteins have been linked to human disease, with LRRK2 in particular being the focus of an intense research effort due to its importance in genetic Parkinson's disease as well as inflammatory bowel disease and leprosy [3]. MASL1 was originally identified in malignant fibrous histiocytomas and has been linked to other forms of cancer [4–7]. It is clear that understanding the cellular and molecular functions of these proteins is an important challenge in several areas of biomedical research.

Based upon bioinformatic analyses, the ROC domain that defines this protein family is predicted to bind guanosine triphosphate (GTP), a property that has been demonstrated for LRRK1, LRRK2 and DAPK1 [8–10]. Functional and structural investigations support a role for GTP binding by the ROC domains of these proteins as well as an involvement in complex formation and in the control of protein function. In the case of DAPK1 and LRRK2, GTP binding has been implicated in the regulation of the kinase activities of these proteins, although the precise mechanism whereby this occurs is unclear [9,11,12]. Structural information obtained from the bacterial *Chlorobium tepidum* ROCO protein suggests that the COR domain functions as a dimerization device to activate ROC GTPase activity [13]. Interestingly, the *C. tepidum* ROCO protein and human MASL1 display conserved domain organization, with N-terminal leucine-rich repeats and a C-terminal ROC-COR bi-domain [13].

MASL1 is unique among the human ROCO proteins in not possessing a clear effector domain, either a kinase (as is the case for LRRK1, LRRK2 and DAPK1) or a death domain (DAPK1). No information regarding the function or structure of this protein is available to date. The aim of this study was therefore to investigate typical ROCO protein features such as GTP binding, GTPase activity and complex formation of MASL1 as well as to identify possible binding partners. These data demonstrate for the first time that MASL1 is a functional GTP binding protein and that complex formation by MASL1 is regulated in a manner dependent on guanosine nucleotide binding.

## Results

### MASL1 is a GTP binding protein

**Figure 2:**
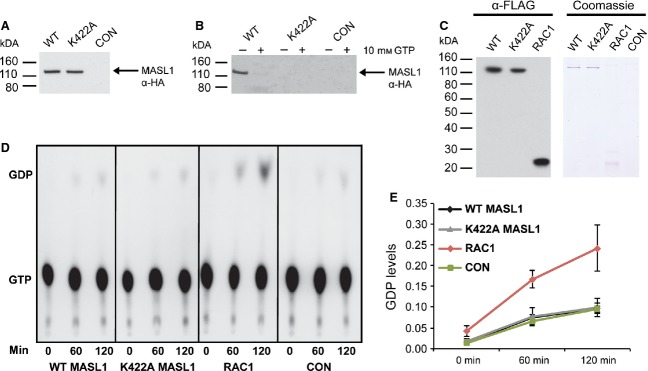
MASL1 – a GTP binding protein. (A) Both wild type and K422A MASL1 expressed equally in HEK293T cells. (B) Overexpression of HA-tagged MASL1 in HEK293T cells was used to determine GTP binding of MASL1. The wild type and K422A forms of the protein were tested for binding to GTP immobilized on sepharose beads with or without a molar excess of GTP. This analysis revealed that wild type MASL1 is a GTP binding protein and that a molar excess of GTP as well as a targeted mutation of the lysine residue at 422 disrupt GTP binding. (C) In order to investigate the ability of MASL1 to hydrolyse GTP both forms of MASL1 and a known GTPase RAC1 were expressed in HEK293T cells. The FLAG-tagged proteins expressed equally (α-FLAG) and could also be eluted in a very pure fashion from FLAG-agarose beads with 150 ng·μL^−1^ FLAG peptide (Coomassie). (D), (E) GTPase activity was observed over a period of 0–120 min displaying activity only by RAC1. No GTPase activity was detected in either form of MASL1 under these experimental conditions. Untransfected HEK293T cells, treated with transfection reagent polyethylenimine (PEI) only, were used as control samples (CON) for all analyses.

To investigate the function of the ROC domain of MASL1, an artificial mutation (K442A), designed to disrupt nucleotide binding, was introduced into the open reading of hemagglutinin (HA) tagged MASL1. This mutation was chosen based on homology to small GTPases and LRRK2, where it has previously been shown to impact upon GTP binding properties [14]. The K422A mutant had no impact on steady state expression of MASL1 (Fig. 2A). Wild type HA-tagged MASL1 was able to bind GTP immobilized on sepharose beads, an interaction that could be disrupted by a molar excess of GTP (10 mm) (Fig. 2B). In contrast, the predicted nucleotide binding deficient form of the protein (K422A MASL1) was incapable of binding GTP, confirming the importance of this residue for GTP binding. To assess whether MASL1 is able to hydrolyse as well as bind GTP, a series of *in vitro* radiometric assays was carried out. As a positive control the small GTPase RAC1 [15] was used. MASL1 (wild type and K422A) and wild type RAC1 could be immunoprecipitated and eluted at similar levels (Fig. 2C). Under the assay conditions used in this experiment, RAC1 displayed GTPase activity whereas neither wild type nor K442A MASL1 was able to hydrolyse GTP above background levels (Fig. 2D,E). Based upon these *in vitro* data, it is impossible to exclude the possibility that MASL1 is capable of hydrolysing GTP in a cellular context with the aid of cofactors.

### Guanosine nucleotides regulate complex formation by MASL1

**Figure 3:**
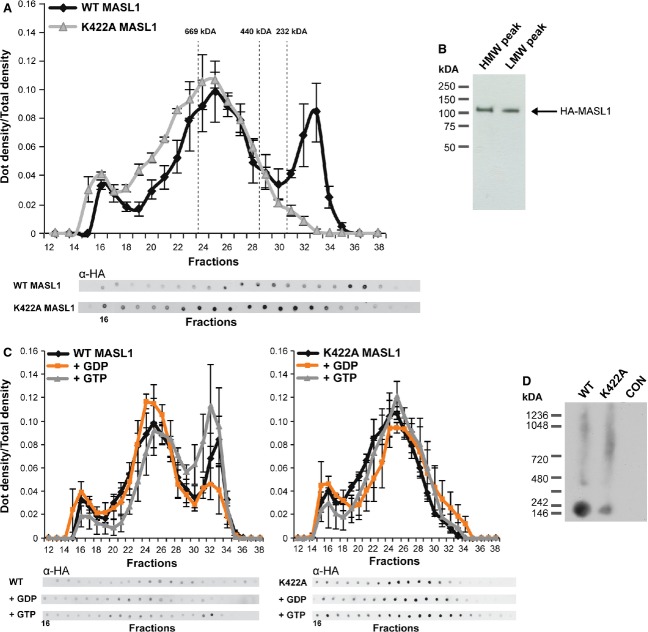
GTP binding properties of MASL1 regulate complex formation. (A) SEC was used to analyse whole cell lysates of MASL1 transfected HEK293T cells. Wild type MASL1 was found in two complexes, an HMW complex and an LMW complex of ∼ 600 and ∼ 200 kDA respectively. In contrast, K422A MASL1 was only found as an HMW complex; representative dot blots are shown. (B) Fractions containing the HMW or LMW complexes were probed for HA showing that full-length MASL1 can be found in both. (C) To investigate the effect of GTP/GDP upon complex formation of MASL1, cell lysates and the SEC column were incubated with 200 μm GTP or GDP prior to analysis. No effect was seen in the K422A mutant. In contrast, wild type MASL1 complexes shifted between HMW and LMW upon GDP or GTP treatment respectively as shown in the graphs. Ratios between HMW and LMW peaks further emphasize this shift: in untreated conditions the ratio between HMW and LMW is 1 : 0.94; upon GDP treatment the equilibrium shifts towards HMW resulting in a ratio of 1 : 0.34; GTP treatment results in a shift towards LMW as seen by a ratio of 1 : 1.28. (D) As an alternative approach to SEC, BN PAGE also showed that wild type MASL1 and the K422A mutant form different complexes. Data are presented as the mean of three individual experiments ± SEM.

Data from a number of studies investigating complex formation by DAPK1 and LRRK2 suggest that the human ROCO proteins form multi-component complexes in a cellular context [10,16–18]. To investigate complex formation by MASL1, size exclusion chromatography (SEC) and blue native (BN) gel electrophoresis were applied to clarified cell lysates of HEK293T cells overexpressing wild type and K422A HA-tagged MASL1. These analyses revealed that wild type MASL1 fractionates as two complexes, a low molecular weight (LMW, ∼ 200 kDa) and a high molecular weight (HMW, ∼ 600 kDa) (Fig. 3A). To verify that the LMW peak was not a degraded product of the HMW complex, SDS/PAGE followed by immunoblotting was carried out on the two chromatographic fractions. As shown in Fig. 3B, both peaks represent full-length proteins. Additionally, wild type and K422A MASL1 were analysed by SEC in the presence of EDTA (5 mm in lysis buffer and SEC running buffer to equilibrate the mobile phase), resulting in a loss of the LMW peak (Fig. S1). These data suggest that the observed shift in MW is due to a disruption of nucleotide binding rather than protein misfolding or degradation. It is notable that loss of nucleotide binding by K422A MASL1 disrupts the LMW peak specifically (Fig. 3A), while it does not impact upon immunoreactivity at ∼ 600 kDa. To examine complex formation using an alternative approach, BN gel electrophoresis was used. In agreement with the SEC data, wild type MASL1 displayed a biphasic distribution with a distinct band at ∼ 200 kDa and a higher molecular mass smear consistent with the ∼ 600 kDa complex observed using SEC. Disruption of nucleotide binding again resulted in a change in apparent molecular mass, with a dramatic reduction in the intensity of the ∼ 200 kDa band (Fig. 3D). To assess whether the loss of guanosine nucleotide binding has an impact on the folding of MASL1, fluorescence spectra were recorded for purified wild type and K422A variants of the protein in the presence and absence of the denaturant guanidine hydrochloride (Fig. S2). Spectra for wild type and K422A forms of MASL1 were comparable, and both underwent a clear shift in spectra upon exposure to 6 m GdHCl suggesting that, under the conditions used in the experiments described above, both forms of MASL1 adopt a native fold.

Based upon the importance of nucleotide binding properties on complex formation by MASL1, the impact of GTP or GDP addition to cell lysates upon the predilection of MASL1 to form LMW or HMW complexes was examined (Fig. 3C). Cell lysates, as well as the mobile phase of the chromatography column, were pre-loaded with 200 μm GTP or GDP. As shown in Fig. 3C, the formation of the LMW complex was modulated by the presence of guanosine nucleotides. Specifically, GDP shifted the equilibrium between LMW and HMW complex towards the latter, whereas incubation with GTP favoured formation of the LMW form of MASL1. Neither GTP nor GDP had any impact on the complex formed by the nucleotide binding dead form of MASL1. As the ability of MASL1 to hydrolyse GTP is still unclear, a SEC run was carried out in the presence of a non-hydrolysable guanosine trinucleotide (GMppCp, Fig. S3). This reveals a similar pattern to that observed for wild type MASL1 in the presence of GTP. Taken together, these data suggest that the guanosine nucleotide binding status of MASL1 has a major impact on complex formation by this protein.

### Heat shock protein 60 (HSP60) interacts with MASL1

**Figure 4:**
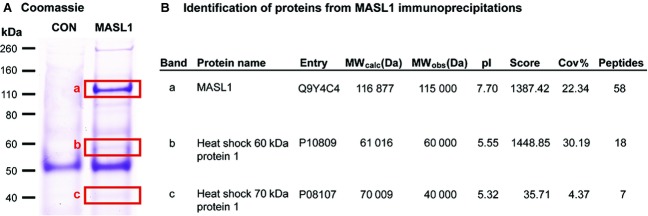
Identification of potential MASL1 interactors. HEK293T cells were transfected with both forms of MASL1 and cell lysates immunoprecipitated with FLAG-agarose beads before being subjected to a series of washing steps with a range of NaCl and Triton X-100 concentrations (see Materials and methods). (A) MASL1 co-purifying bands, not seen in CON samples (insets a, b, c), were selected for analysis. (B) Excised bands were subjected to MALDI-QTOF MS and identified as (a) MASL1, (b) HSP60 and (c) HSP70. While HSP60 co-purified at the predicted molecular weight, HSP70 was found in a band with the incorrect molecular weight and a low correlation score (∼ 4%). HSP60 was found in two out of three MS experiments carried out.

**Figure 5:**
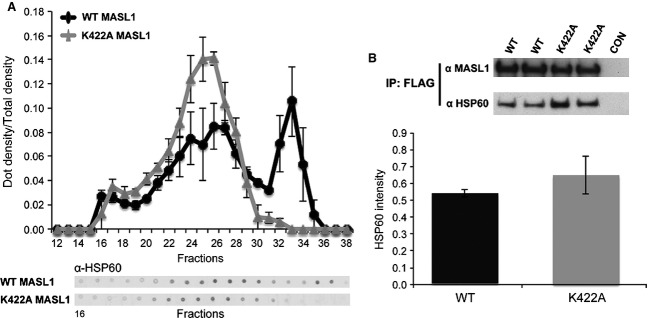
Interaction of MASL1 and HSP60. (A) Fractions obtained from SEC analysis of wild type and K422A MASL1 were probed for HSP60, showing a similar distribution to MASL1 with no HSP60 present in the LMW fraction. Representative dot-blot images also show that HSP60 expression follows the same pattern as MASL1. (B) To verify these findings we also analysed HSP60 co-immunoprecipitation with FLAG-tagged MASL1. Wild type and K422A MASL1 co-immunoprecipitated HSP60 at similar levels. Data are presented as the mean of three individual experiments ± SEM.

The presence of MASL1 in two cellular complexes of ∼ 600 kDa and ∼ 200 kDa suggests that MASL1 may form an active complex with other proteins in the cell. To identify such potential binding partners, HA-tagged MASL1 protein overexpressed in HEK293T cells was immunoprecipitated. Co-purifying proteins were isolated by SDS/PAGE (Fig. 4A) and the corresponding bands were subjected to mass spectrometry analysis (Fig. 4B). A representative image (Fig. 4A) shows that MASL1 (band a) was successfully immunoprecipitated as well as additional bands representing possible interactors/binding partners, which were not present in the control condition (untransfected). Two major bands of ∼ 60 and ∼ 40 kDa were analysed and identified as HSP60 and HSP70 respectively (Fig. 4B). The band at 60 kDa (band b) was identified as HSP60 and displayed a high correlation score (30%). The band at 40 kDa (band c) was identified as HSP70 and displayed a low correlation score. Due to the divergence in the relative molecular mass of this band and the predicted mass of HSP70, in combination with the low correlation score, subsequent experiments focused on HSP60. To validate these mass spectrometry findings, the SEC fractions of HEK293T cell lysates expressing MASL1 (either wild type or K422A) were assessed for the presence of HSP60. These data reveal HSP60 as being present in the same fractions as MASL1 (Fig. 5A). In co-immunoprecipitation experiments followed by immunoblot for HSP60, MASL1 (both wild type and mutant) was found to co-purify with HSP60 (Fig. 5B).

### Cellular localization of MASL1

**Figure 6:**
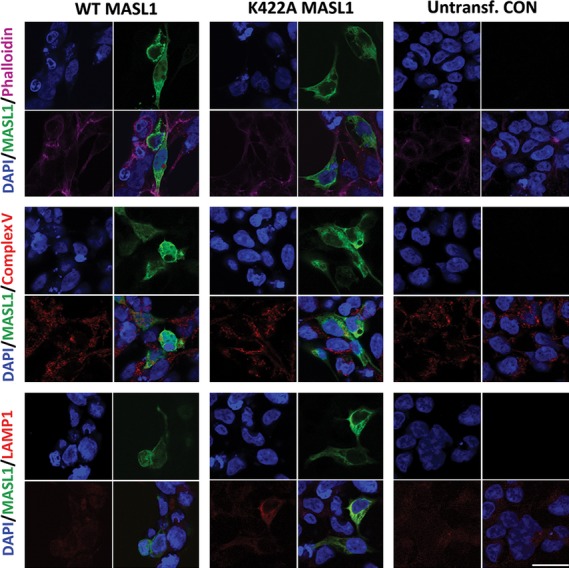
Cytosolic localization of MASL1. Wild type and K422A MASL1 expressed at equal levels in HEK293T cells. Despite the lysine mutation at 422 no changes in the expression pattern were observed. Co-expression with the cytoskeletal marker phalloidin, the mitochondrial marker complex V and the lysosomal marker LAMP1 were analysed. No apparent co-localization was observed with the markers used in this study. Scale bar, 10 μm.

Given the impact of guanosine nucleotide occupancy on complex formation by MASL1, the cellular localization of wild type and K422A MASL1 was assessed by immunocytochemistry. To achieve this, HEK293T cells transfected with HA-tagged wild type and K422A forms of MASL1 were analysed by immunocytochemistry and confocal microscopy. Both wild type and K422A MASL1 were expressed at similar levels and displayed broad cytoplasmic localization (Fig. 6). ROCO proteins have been suggested to participate in vesicle cycling; in particular LRRK2 regulates synaptic vesicle trafficking and LRRK1 epidermal growth factor receptor trafficking [19–21]. To determine whether MASL1 preferentially associates with any specific organelles/structures within the cell, co-localization studies were performed with the lysosomal marker LAMP1, the cytoskeletal marker phalloidin and complex V as a marker of mitochondria. Although both forms of MASL1 occasionally co-localized with the lysosomal marker LAMP1, no consistent co-localization with the markers used in this study was observed (Fig. 6).

### Cytotoxicity of MASL1 in HEK293T cells

**Figure 7:**
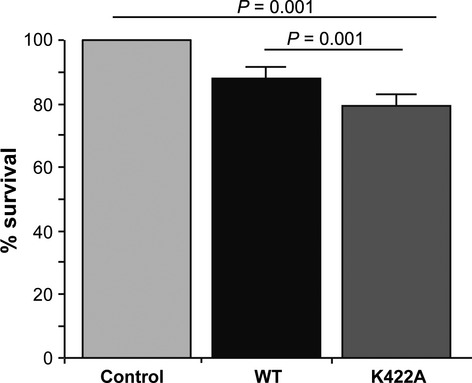
Increased cellular toxicity in the GTP dead form of MASL1. Expression of wild type and K422A MASL1 resulted in significantly increased cellular toxicity compared with untransfected control cells. In addition, cellular toxicity was more severe in the GTP dead form of the protein compared with wild type MASL1. Data are presented as the mean of three individual experiments ± SEM. One-way ANOVA followed by the Bonferroni *post hoc* test was used to determine potential statistical significance, with *P* values ≤ 0.05 considered significant.

**Figure 8:**
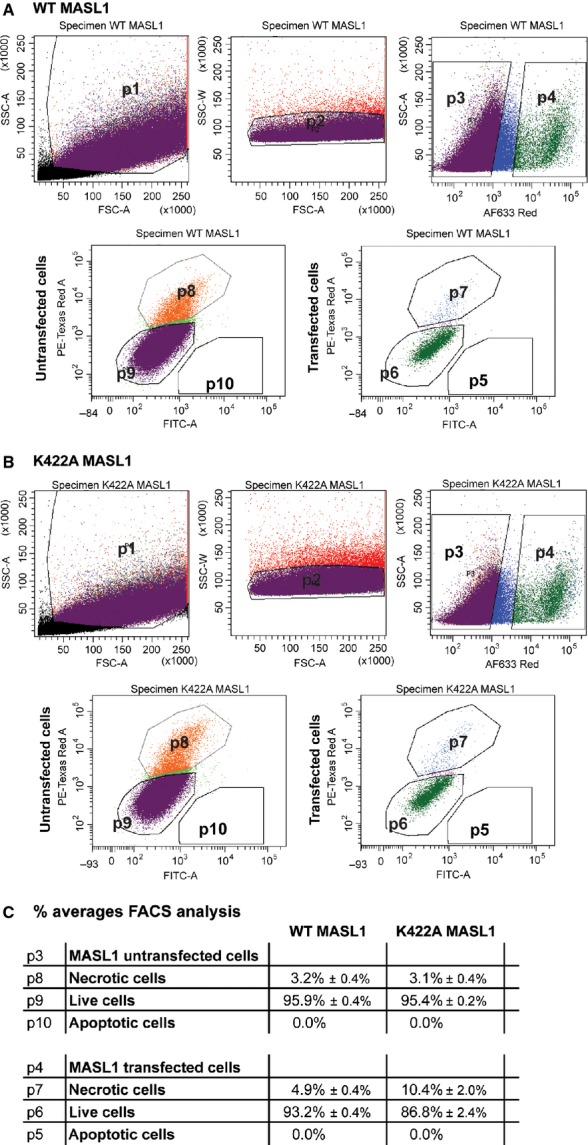
Cellular toxicity upon MASL1 expression. HEK293T cells were transfected with wild type and K422A MASL1. A cell death staining kit detecting apoptotic, live and necrotic cells enabled the analysis of cell death via FACS. Cells were sorted by size and complexity prior to separating untransfected (p3) from MASL1 transfected (p4) cells. (A) Wild type MASL1 transfection resulted in ∼ 3% and ∼ 5% necrotic cells in untransfected or transfected cells respectively. (B) K422A MASL1 also led to ∼ 3% necrotic cells in the untransfected cell population but resulted in ∼ 10% necrotic cells in K422A positive cells. (C) FACS analysis of *n *=* *3 per wild type and K422A MASL1 transfected cells displayed as the average of three experiments in percentage ± SEM.

Overexpression of the ROCO proteins DAPK1 and LRRK2 is associated with cytotoxicity in cellular models [22–24]. In common with both of these proteins, MASL1 has been implicated in cancer suggesting a possible role in the control of cell death or proliferation. To examine cellular toxicity of MASL1 upon transfection in HEK293T cells, two approaches were used: a 3-(4,5-dimethylthiazol-2-yl)-2,5-diphenyltetrazolium bromide (MTT) assay and fluorescence-activated cell sorting (FACS) to allow identification of viable, necrotic and apoptotic cells. Expression of wild type MASL1 resulted in a significant decrease in viable cells compared with controls, an effect that was exacerbated by the expression of the nucleotide binding dead form of the protein (Fig. 7). To confirm these observations with an independent technique, FACS analysis of cells transfected with MASL1 was carried out. Approximately 5000 transfected cells were analysed per sample (*n *=* *3 per genotype). Untransfected control cells were used to set up gating of transfected cells (p4) (Fig. S4). Analysis of MASL1 positive cells showed a similar trend towards increased cell death in K422A MASL1 transfected cells (p4) as observed with the MTT assay (Fig. 8). This increase predominantly consisted of necrotic cells (Fig. 8B,C). No difference of either apoptosis or necrosis could be observed in the untransfected cell population (p3) or with control cells (Fig. S4).

## Discussion

This study provides an insight into the biology of MASL1, the smallest member of the human ROCO protein family. The data presented here demonstrate that MASL1 possesses an ROC domain that is capable of binding GTP, and that guanosine binding properties of MASL1 are important for complex formation and for the cytotoxic properties of this protein.

Two approaches were used to determine the GTP binding properties of MASL1. First, a competitive GTP binding assay in which MASL1 was incubated with or without GTP free in solution along with immobilized GTP on sepharose beads showed that the wild type protein is able to bind GTP and that this interaction is competed away by incubation with a molar excess of GTP. Second, a targeted genetic disruption of a conserved lysine (K422) important for nucleotide binding identified by sequence alignment with DAPK1 and LRRK2 resulted in complete loss of GTP binding. These data are consistent with results obtained for the other human ROCO proteins.

Protein binding to GTP immobilized on sepharose beads is a useful technique to assess the ability of MASL1 to bind GTP; however, it does not provide any information about the ability of this protein to hydrolyse GTP. MASL1 GTPase activity was therefore assessed using previously published protocols [14]. Using the small GTPase RAC1 as a positive control, FLAG-tagged MASL1 free in solution displayed no detectable GTPase activity above background. While these findings suggest that MASL1 has very low endogenous GTPase activity, it is possible that cofactors such as GTPase activating proteins are required for MASL1 to hydrolyse GTP at detectable rates *in vitro*.

The loss of GTP binding has the potential to disrupt the stability of MASL1; however, analysis of steady state expression levels of the wild type and K422A forms of the protein demonstrated that both forms have equivalent expression levels, suggesting that loss of nucleotide binding does not result in a major alteration in folding. This is further supported by SEC analysis in the presence of EDTA (Fig. S1), which demonstrates that disruption of nucleotide binding results in similar changes in complex formation of MASL1 as observed for the K422A mutant. Fluorescence data from the wild type and K422A forms of the protein indicate that both adopt a folded conformation when purified and react in an identical fashion upon denaturation. It is also notable that the K422A mutant protein displayed a similar pattern of cellular localization compared with wild type MASL1, suggesting that the nucleotide binding dead form of the protein is not destabilized to the point where it is targeted for degradation. One possible consequence of amino acid substitutions impacting on active site binding such as the K422A mutation is an increased co-expression of chaperones such as HSP60 in order to aid protein folding. However, when co-immunoprecipitation of HSP60 was examined no significant difference was observed between wild type and K422A MASL1, suggesting that the lysine to alanine mutation at 422 does not destabilize the protein.

The paucity of data relating to MASL1 precludes direct comparisons with other MASL1 studies. Such comparisons are possible, however, with the other members of the ROCO protein family – providing an opportunity to examine whether MASL1 shares features with its close paralogues. A number of studies have demonstrated that there is a close link between the enzymatic function or nucleotide binding of the ROCO proteins and the complexes that they form, and that a disruption of guanosine nucleotide binding can impair dimerization as is the case for LRRK2 [17,25]. In contrast to LRRK2, loss of guanosine nucleotide binding does not appear to disrupt dimerization of DAPK1 but more probably controls the equilibrium between dimeric or multimeric forms of DAPK1 or controls recruitment of cofactors [10,12]. The SEC data from the experiments described above reveal that MASL1 can be found in two distinct complexes, one of ∼ 600 kDa and a smaller one with an apparent molecular mass of ∼ 200 kDa (Fig. 3). While both wild type and mutant forms of MASL1 eluted at ∼ 600 kDa, only the wild type form of MASL1 (i.e. able to bind guanosine nucleotides) eluted at ∼ 200 kDa. Although the precise nature of the two complexes remains to be determined, these data support a model where the LMW complex represents monomeric MASL1 associated with a binding partner/partners of approximately 50–80 kDa, as a dimeric MASL1 complex should migrate at a higher apparent molecular mass of > 220 kDa. These data appear to conflict with a recent model for ROCO protein function [26]; however, an important caveat to the data reported above is the low resolution of both SEC and BN analyses of cellular complexes and so it is impossible to exclude the possibility that this lower peak consists in part of dimeric MASL1. This highlights the need to derive more detailed structural information for the ROCO proteins, including MASL1.

An affinity purification coupled with mass spectrometry analysis revealed that MASL1 strongly interacts with HSP60. Although the interaction could be influenced by the overexpression of MASL1, this result suggests that MASL1 probably requires the aid of chaperones to be correctly folded. It is noteworthy that LRRK2 has been shown to interact with a chaperone, HSP90, and disruption of this interaction results in substantial loss of LRRK2 stability [27]. Further studies are required to determine whether additional interacting proteins are responsible for the observed complex formation between wild type and the nucleotide binding dead form of MASL1.

Very little is known about the cellular role of MASL1. Two recent publications have highlighted a potential role in regulating inflammatory response, and data from both DAPK1 and LRRK2 suggest that these proteins may be involved in pathways regulating cell death/proliferation. In light of the link between MASL1 and cancer, the ability of this protein to bind guanosine nucleotides (and the impact of this upon its biology and the complexes that MASL1 forms) may be important for processes such as proliferation, cell death or inflammatory signalling. Based upon previous experiments linking ROC domain biology to the toxicity of LRRK2 and DAPK1, the impact of overexpressing MASL1 and the K422A mutant of MASL1 was evaluated. Wild type MASL1 displayed a cytotoxic impact in HEK293T cells as measured by MTT assay – a toxicity that was accentuated to a small but significant extent by the loss of guanosine nucleotide binding. These data are in contrast to results for LRRK2 and DAPK1, where loss of guanosine nucleotide binding decreases the cytotoxic phenotype observed upon overexpression of these proteins. This result was further supported by data from FACS analysis of transfected cells. Following on from these data, it is noteworthy that the cell death induced by MASL1 expression is almost exclusively necrotic, providing a potential insight into the pathways that MASL1 is involved in. Although expression of wild type MASL1 alone results in cytotoxicity (as is observed for both LRRK2 and DAPK1), these data support a role for the switch from GTP to GDP active site occupancy in modulating this toxic impact, with loss of guanine nucleotide binding (and shift to the higher molecular mass complex) accentuating toxicity.

**Figure 9:**
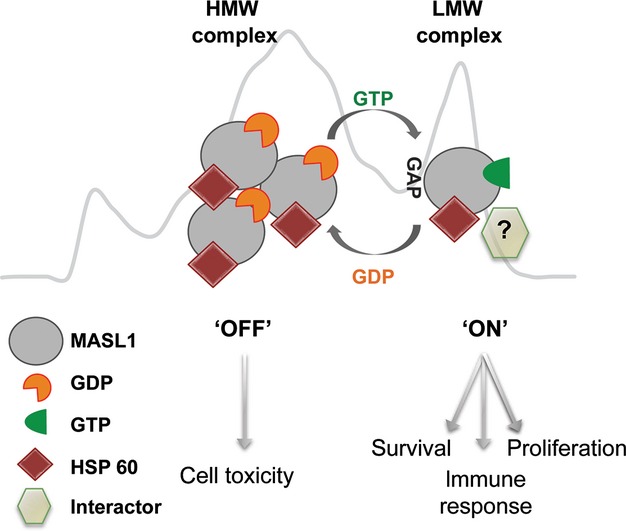
Complex formation, nucleotide binding and interactors – a working model for MASL1. The exact biological function of MASL1 is currently unknown. The data included in this study describe basic MASL1 biochemical properties such as GTP binding, complex formation and possible interactors. Based on this work a hypothetical model for MASL1 can be proposed. MASL1, like GTP binding proteins in general, can be found in two conformational states, one being ‘on’ (GTP bound) and the other ‘off’ (GDP bound). This model proposes that MASL1 in the ‘on’ state impacts upon a binding partner/interactor (possibly including HSP60) via an effector domain such as a kinase domain.

Taken together, the data from this study are consistent with a model for MASL1 function where guanosine nucleotide binding/occupancy controls complex formation and the downstream consequences of MASL1 activity (Fig. 9). Based upon the conserved role of guanosine nucleotide binding proteins as molecular switches within the cell, MASL1 may act to regulate cell death/proliferation based upon its GTP binding. One potential scenario is that the GTP-bound state represents the ‘on’, or active, state with MASL1 competent to bind its cellular effector(s), while losing the affinity for its effector in the GDP-bound ‘off’ state results in accumulation within the HMW pool. In this scenario, the HMW complex could represent an inactive, readily releasable pool of oligomeric MASL1. Future information on the identity of effector proteins/binding partners could provide important clues to the physiological function of MASL1, as well as presenting opportunities to test whether the model in Fig. 9 is correct. For example, the identification of candidate guanine nucleotide exchange factors or GTPase activating proteins that interact with MASL1 may shed light on how the transition from one complex to another is managed within the cell. It should be noted, however, that basal toxicity is observed independent of alterations in the ROC domain of MASL1, and it is possible that cytoxicity linked to MASL1 does not rely on the GTP/GDP-bound status of the holoprotein and is independent of the ROC domain. Further investigations into MASL1 function will reveal which scenario is correct.

With regard to the cellular role of MASL1, it is of interest that a subset of human leucine-rich repeat proteins function as pathogen responsive genes in primary macrophages, and MASL1 in particular was shown to regulate Toll-like receptor signalling and the Raf/MEK/ERK signalling pathway in CD34(+) cells [28,29]. As LRRK2 has also been robustly linked to immune function [30–32], one intriguing possibility is that immune response regulation represents a converging function of human ROCO proteins and that alteration of their immune-related function (i.e. mutations) leads to disease [33].

In summary, the data presented in this study represent the first report of the biochemical and cellular properties of MASL1. The results demonstrate that MASL1 is a GTP binding protein and that its GTP binding properties act to regulate complex formation and cytotoxicity *ex vivo*. Further studies are required to determine the exact pathways that this protein is involved in, and to understand its basic cellular function and possible contribution towards pathology. Importantly, a more detailed understanding of this so far little studied protein has the potential to contribute to a greater comprehension of how the ROCO proteins function in a cellular context.

## Materials and methods

### Constructs

HA and FLAG epitope tagged wild type MASL1 constructs were obtained from Genecopoeia (http://www.genecopoeia.com). To generate mutant forms of MASL1 site directed mutagenesis was carried out using the QuikChange II mutagenesis kit (Agilent, Santa Clara, CA, USA) according to the manufacturer's instructions. Constructs were sequen-ced prior to use and primer sequences used for the mutagenesis are available from the authors upon request.

### Cell culture and MASL1 expression

HEK293T cells were obtained from ATCC® (line identifier CRL-1573) and grown under standard tissue culture conditions at 37 °C in 5% CO_2_ in DMEM (Life Technologies, Grand Island, NY, USA) supplemented with 10% fetal bovine serum (Life Technologies). To transfect cells 20 μg of plasmid DNA was dissolved in 500 μL DMEM before 40 μL polyethylenimine (PEI, Polysciences, Warrington, PA, USA) was added and the solution was mixed and left to incubate for a minimum of 15 min at room temperature. The DNA/PEI solution was then added to cells in 10–15 cm Petri dishes with experimental procedures being carried out 48 h thereafter. PEI solution treated HEK293T cells were used throughout this study as control samples. All incubations, washes and other treatments were kept consistent between MASL1 and control samples.

### GTP pulldown assay

Forty-eight hours after transfection HEK293T cells were washed twice with ice-cold phosphate buffered saline (NaCl/P_i_) prior to lysis. This was achieved by harvesting cells in cell lysis buffer (Cell Signaling Technology, Danvers, MA, USA) containing 1 ×  complete protease inhibitor cocktail (Roche, Indianapolis, IN, USA). Subsequently cell lysates were rotated for 30 min at 4 °C and then spun at 10 000 ***g*** for 10 min to remove cell debris. Next cell lysates were incubated with 35 μL sepharose beads for 30 min at 4 °C and rotation. Finally, cell lysates were again centrifuged at 10 000 ***g***, 4 °C for 10 min to remove sepharose beads, with the clarified lysates used in subsequent experiments. GTP-conjugated sepharose beads (Sigma, St Louis, MO, USA) were washed with lysis buffer and incubated for 1 h at 4 °C and rotation with 0.1 mg·mL^−1^ BSA. GTP beads were then added to cell lysates for 2 h at 4 °C and rotation ± 10 mm GTP (Sigma) before beads were washed three times with lysis buffer and denatured in 4 ×  SDS loading buffer (NuPage SDS sample buffer, Life Technologies) with 5% β-mercaptoethanol at 100 °C for 10 min. Denatured samples were loaded onto 4–12% Bis Tris gels (Life Technologies). Simultaneously, to verify MASL1 expression 10 μg per sample as determined by bicinchoninic acid assay (ThermoFisher, Rockland, IL, USA) according to the manufacturer's instructions were also loaded onto Bis Tris gels (Life Technologies). Proteins were then transferred to poly (vinylidene difluoride) membrane (Millipore, Billerica, MA, USA), blocked with 5% milk in NaCl/P_i_-Tween (NaCl/P_i_ + 1% Tween-20) and probed with primary antibodies at 4 °C overnight (1 : 2000 rat anti-HA, Roche; 1 : 5000 mouse anti-β-actin, Sigma). The next day membranes were washed three times in NaCl/P_i_-Tween and incubated for 1 h with secondary antibodies in 5% milk in NaCl/P_i_-Tween at room temperature followed by another three washes and incubation with Pierce ECL substrate. Finally membranes were exposed to Kodak biomax films (Kodak, Rochester, NY, USA) and developed on a Kodak developer according to the manufacturer's instructions.

### GTPase assay

Cell lysis was carried out as described above with cell lysis buffer (Cell Signaling Technology). Wild type MASL1, K422A MASL1 and RAC1 supernatants were incubated with anti-FLAG beads (Sigma) overnight at 4 °C. The next day beads were washed 2 ×  in each of five wash buffers (20 mm Tris, 150–500 mm NaCl, 0.02–1% Triton X-100) before being eluted in elution buffer (20 mm Tris, 150 mm NaCl, 0.02% Triton X-100) with 150 ng·μL^−1^ FLAG peptide (Sigma) for 45 min at 4 °C. Eluted protein was then incubated with assay buffer (20 mm Hepes, pH 7.2, 2 mm MgCl_2_, 1 mm dithiothreitol and 0.05% BSA) and [α^32^P]GTP (5 μCi; Perkin Elmer, Waltham, MA, USA) was added to each reaction. Samples were incubated at 37 °C with vigorous shaking and 2 μL aliquots were removed at time points from 0 to 120 min and spotted onto TLC plates (Sigma). Samples were then subjected to rising TLC under 1 m formic acid and 1.2 m LiCl. After drying the plates they were exposed to Kodak biomax films overnight and developed the following day using a Kodak developer. The remaining samples were denatured, run on SDS/PAGE, transferred onto poly(vinylidene difluoride) membranes and immunoblotted as described above.

### Intrinsic fluorescence

Fluorescence emission spectra were recorded on a Cary Eclipse fluorescence spectrophotometer (Agilent Technologies) using the Cary Eclipse program. Sample measurements were carried out using an optical path length of 10 mm. Fluorescence spectra were obtained using an excitation wavelength of 288 nm, with an excitation bandwidth of 5 nm. Emission spectra were recorded between 300 and 400 nm at a scan rate of 30 nm·s^−1^. Spectra were acquired using 100 nm proteins in 20 mm Tris/HCl buffer (pH 7.5), 150 mm NaCl and 0.02% Tween-20.

### Immunocytochemistry

To visualize expression of MASL1, transfected HEK293T cells were grown on glass coverslips (VWR, Radnor, PA, USA) for 48 h after transfection before being fixed with 4% PFA at room temperature for 10 min. Fixed cells were then blocked for 30 min in 15% normal goat serum (NGS) in NaCl/P_i_ + 0.1% Triton X-100 before incubation with primary antibodies in 10% NGS in NaCl/P_i_ + 0.1% Triton X-100 (1 : 2000 rat anti-HA, Roche; 1 : 1000 mouse anti-LAMP1; 1 : 500 rabbit anti-complex V) at 4 °C overnight. Next cells were incubated with fluorescently labelled secondary antibodies in 10% NGS in NaCl/P_i_ + 0.1% Triton X-100 (goat anti-rat 488, goat anti-mouse IgG1 568; goat anti-rabbit 568) before being counterstained with DAPI at 1 : 2000 in NaCl/P_i_. To detect F-actin Alexa Fluor^**®**^ 633 phalloidin was used at 1 : 300 (Life Technologies) before cells were mounted onto uncoated glass slides (VWR) using Fluoromount G mounting medium (Southern Biotech, Birmingham, AL, USA). Images were obtained on an LSM 710 confocal microscope and compiled using Adobe Photoshop.

### Size exclusion chromatography

MASL1 transfected HEK293T cell lysates obtained as described above were injected and separated on a Superose 6 10/300 column (GE Healthcare, Little Chalfont, Buckinghamshire, UK). The column was pre-equilibrated with buffer (20 mm Tris/HCL, pH 7.5, 150 mm NaCl and 0.07% Tween-20 or with the same buffer containing 200 μm GTP, GDP or GMppCp) and used at a flow rate of 0.5 mL·min^−1^. Elution volumes of standards were 12 mL for thyreoglobin (669 kDA), 14 mL for ferritin (440 kDA), 15.5 mL for catalase (232 kDA) and 17 mL for aldolase (158 kDA). Elution fractions were collected and analysed using dot blots. For the analysis in the presence of guanosine nucleotides the Superose 6 10/300 column was pre-equilibrated with buffer (as mentioned above) containing 200 μm GTP, GDP or GMppCp for 60 min. Cell lysates were incubated with the same concentration of nucleotides prior to analysis by SEC.

### Dot blot

1 μL of each collected SEC fraction was spotted onto a nitrocellulose membrane. Once dried the membrane was blocked in NaCl/P_i_-Tween with 5% milk for 1 h before being incubated with 1 : 2000 rat anti-HA and secondary anti-rat (Sigma) both in 5% milk. Membranes were then developed as described above.

### Mass spectrometry analysis

MS analysis was carried out by the Biological Mass Spectrometry Centre at the Institute of Child Health, University College London (http://www.ucl.ac.uk/ich/services/labservices/mass_spectrometry). For this analysis MASL1 transfected HEK293T cells were lysed, immunoprecipitated and washed as described above (GTPase assays) before denatured samples were loaded and run on 4–12% Bis Tris gels (Life Technologies), stained with Imperial Protein Stain (Thermo Scientific) and excised from gels (Fig. 4A) for MS analysis by MALDI-QTOF MS (Waters Corporation, Milford, MA, USA).

### Cell toxicity assay

Forty-eight hours post-transfection of HEK293T cells with MASL1 (wild type and mutant forms) MTT (Sigma Aldrich) was added at a final concentration of 500 μg·mL^−1^ for 3 h. The medium was then discarded before formazan crystals, accumulated within energetically active cells, were dissolved in pure dimethylsulfoxide. The assay was carried out in 96-well plates, which were then analysed in a multi-well plate reader measuring the absorbance of every single well at a wavelength of 570 nm. Results are shown as percentage cell viability after transfection in comparison with transfection reagent only treated cells.

### Fluorescence-activated cell sorting

To establish cellular toxicity of wild type and K422A MASL1 in HEK293T cells an apoptotic/necrotic/healthy cells detection kit (Promokine, Heidelberg, Germany) was used, following the manufacturer's instructions. To detect MASL1 transfected cells a fluorescently labelled FLAG-tag antibody (DYKDDDDK-tag antibody, Alexa Fluor 647 conjugate; Cell Signaling) was used. Cells were stained for 30 min in NaCl/P_i_ containing 0.1% Triton X-100 with FLAG-tag antibody at 1 : 500. Post staining cells were resuspended in cold NaCl/P_i_ containing 0.1 mm EDTA to minimize clumping and passed through a cell strainer (70 μm) (BD Biosciences, San Jose, CA, USA). Cells were sorted on a BD Bioscience FACS LSRII running facsdiva6.3 software. Fluorescently labelled cells were excited using the red laser (560 nm), with emission analysed using the PE PMT via a 505 long pass and 525/50 band pass filter, the green laser (488 nm) and analysed using the FITC PMT via a 505 long pass and a 525/50 band pass filter, or a violet (405 nm) laser and the Pacific Blue PMT with a 450/50 band pass filter. Debris, doublets and dead cells were excluded from analysis (Fig. 8, p2 represents all cells analysed). Approximately 27 000 FLAG positive cells were analysed for both wild type and K422A MASL1.

### Statistical analysis

spss software (IBM, Armonk, NY, USA) was used for statistical comparison. To assess potential significant differences one-way analysis of variance analysis (ANOVA) with Bonferroni *post hoc* correction was used. Significance was considered to be < 0.05.

## Abbreviations

BN: blue native
COR: C-terminal of ROC
DAPK1: death-associated protein kinase 1
FACS: fluorescence-activated cell sorting
HA: hemagglutinin
HMW: high molecular weight
HSP: heat shock protein
LMW: low molecular weight
LRRK1: leucine-rich repeat kinase 1
LRRK2: leucine-rich repeat kinase 2
MASL1: malignant fibrous histiocytoma amplified sequences with leucine-rich tandem repeats 1
MTT: 3-(4,5-dimethylthiazol-2-yl)-2,5-diphenyltetrazolium bromide
ROC: Ras of complex proteins
SEC: size exclusion chromatography

## Supporting information

**Fig. S1.** Nucleotide binding regulates complex formation. **Fig. S2.** Representative fluorescence spectra of purified wild type and K422A MASL1 in the presence and absence of 6 m GdHCl using an excitation wavelength of 280 nm. **Fig. S3.** SEC analysis of wild type MASL1 in the presence of 200 µm GTP or GMppCp. **Fig. S4.** FACS analysis quantification.

## References

[1] Bosgraaf L, Van Haastert PJ (2003). Roc, a Ras/GTPase domain in complex proteins. Biochim Biophys Acta.

[2] Lewis PA (2009). The function of ROCO proteins in health and disease. Biol Cell.

[3] Lewis PA, Manzoni C (2012). LRRK2 and human disease: a complicated question or a question of complexes?. Science Signaling.

[4] Sakabe T, Shinomiya T, Mori T, Ariyama Y, Fukuda Y, Fujiwara T, Nakamura Y, Inazawa J (1999). Identification of a novel gene, MASL1, within an amplicon at 8p23.1 detected in malignant fibrous histiocytomas by comparative genomic hybridization. Cancer Res.

[5] Tagawa H, Karnan S, Kasugai Y, Tuzuki S, Suzuki R, Hosokawa Y, Seto M (2004). MASL1, a candidate oncogene found in amplification at 8p23.1, is translocated in immunoblastic B-cell lymphoma cell line OCI-LY8. Oncogene.

[6] Yang S, Jeung HC, Jeong HJ, Choi YH, Kim JE, Jung JJ, Rha SY, Yang WI, Chung HC (2007). Identification of genes with correlated patterns of variations in DNA copy number and gene expression level in gastric cancer. Genomics.

[7] Weng WH, Wejde J, Ahlen J, Pang ST, Lui WO, Larsson C (2004). Characterization of large chromosome markers in a malignant fibrous histiocytoma by spectral karyotyping, comparative genomic hybridization (CGH), and array CGH. Cancer Genet Cytogenet.

[8] Korr D, Toschi L, Donner P, Pohlenz HD, Kreft B, Weiss B (2006). LRRK1 protein kinase activity is stimulated upon binding of GTP to its Roc domain. Cell Signal.

[9] Ito G, Okai T, Fujino G, Takeda K, Ichijo H, Katada T, Iwatsubo T (2007). GTP binding is essential to the protein kinase activity of LRRK2, a causative gene product for familial Parkinson's disease. Biochemistry.

[10] Carlessi R, Levin-Salomon V, Ciprut S, Bialik S, Berissi H, Albeck S, Peleg Y, Kimchi A (2011). GTP binding to the ROC domain of DAP-kinase regulates its function through intramolecular signalling. EMBO Repts.

[11] Taymans JM, Vancraenenbroeck R, Ollikainen P, Beilina A, Lobbestael E, De Maeyer M, Baekelandt V, Cookson MR (2011). LRRK2 kinase activity is dependent on LRRK2 GTP binding capacity but independent of LRRK2 GTP binding. PLoS ONE.

[12] Jebelli JD, Dihanich S, Civiero L, Manzoni C, Greggio E, Lewis PA (2012). GTP binding and intramolecular regulation by the ROC domain of death associated protein kinase 1. Sci Rep.

[13] Gotthardt K, Weyand M, Kortholt A, Van Haastert PJ, Wittinghofer A (2008). Structure of the Roc-COR domain tandem of *C. tepidum*, a prokaryotic homologue of the human LRRK2 Parkinson kinase. EMBO J.

[14] Lewis PA, Greggio E, Beilina A, Jain S, Baker A, Cookson MR (2007). The R1441C mutation of LRRK2 disrupts GTP hydrolysis. Biochem Biophys Res Commun.

[15] Menard L, Tomhave E, Casey PJ, Uhing RJ, Snyderman R, Didsbury JR (1992). Rac1, a low-molecular-mass GTP-binding-protein with high intrinsic GTPase activity and distinct biochemical properties. Eur J Biochem/FEBS J.

[16] Klein CL, Rovelli G, Springer W, Schall C, Gasser T, Kahle PJ (2009). Homo-and heterodimerization of ROCO kinases: LRRK2 kinase inhibition by the LRRK2 ROCO fragment. J Neurochem.

[17] Greggio E, Zambrano I, Kaganovich A, Beilina A, Taymans JM, Daniels V, Lewis P, Jain S, Ding J, Syed A (2008). The Parkinson disease-associated leucine-rich repeat kinase 2 (LRRK2) is a dimer that undergoes intramolecular autophosphorylation. J Biol Chem.

[18] Civiero L, Vancraenenbroeck R, Belluzzi E, Beilina A, Lobbestael E, Reyniers L, Gao F, Micetic I, De Maeyer M, Bubacco L (2012). Biochemical characterization of highly purified leucine-rich repeat kinases 1 and 2 demonstrates formation of homodimers. PLoS ONE.

[19] Piccoli G, Condliffe SB, Bauer M, Giesert F, Boldt K, De AstisS, Meixner A, Sarioglu H, Vogt-Weisenhorn DM, Wurst W (2011). LRRK2 controls synaptic vesicle storage and mobilization within the recycling pool. J Neurosci.

[20] Biskup S, Moore DJ, Celsi F, Higashi S, West AB, Andrabi SA, Kurkinen K, Yu SW, Savitt JM, Waldvogel HJ (2006). Localization of LRRK2 to membranous and vesicular structures in mammalian brain. Ann Neurol.

[21] Hanafusa H, Ishikawa K, Kedashiro S, Saigo T, Iemura S, Natsume T, Komada M, Shibuya H, Nara A, Matsumoto K (2011). Leucine-rich repeat kinase LRRK1 regulates endosomal trafficking of the EGF receptor. Nat Commun.

[22] Greggio E, Lewis PA, van der Brug MP, Ahmad R, Kaganovich A, Ding J, Beilina A, Baker AK, Cookson MR (2007). Mutations in LRRK2/dardarin associated with Parkinson disease are more toxic than equivalent mutations in the homologous kinase LRRK1. J Neurochem.

[23] Smith WW, Pei Z, Jiang H, Dawson VL, Dawson TM, Ross CA (2006). Kinase activity of mutant LRRK2 mediates neuronal toxicity. Nat Neurosci.

[24] Cohen O, Feinstein E, Kimchi A (1997). DAP-kinase is a Ca^2+^/calmodulin-dependent, cytoskeletal-associated protein kinase, with cell death-inducing functions that depend on its catalytic activity. EMBO J.

[25] Berger Z, Smith KA, Lavoie MJ (2010). Membrane localization of LRRK2 is associated with increased formation of the highly active LRRK2 dimer and changes in its phosphorylation. Biochemistry.

[26] Gasper R, Meyer S, Gotthardt K, Sirajuddin M, Wittinghofer A (2009). It takes two to tango: regulation of G proteins by dimerization. Nat Rev Mol Cell Biol.

[27] Wang L, Xie C, Greggio E, Parisiadou L, Shim H, Sun L, Chandran J, Lin X, Lai C, Yang WJ (2008). The chaperone activity of heat shock protein 90 is critical for maintaining the stability of leucine-rich repeat kinase 2. J Neurosci.

[28] Ng AC, Eisenberg JM, Heath RJ, Huett A, Robinson CM, Nau GJ, Xavier RJ (2011). Human leucine-rich repeat proteins: a genome-wide bioinformatic categorization and functional analysis in innate immunity. Proc Natl Acad Sci U S A.

[29] Kumkhaek C, Aerbajinai W, Liu W, Zhu J, Uchida N, Kurlander R, Hsieh MM, Tisdale JF, Rodgers GP (2013). MASL1 induces erythroid differentiation in human erythropoietin-dependent CD34 +  cells through the Raf/MEK/ERK pathway. Blood.

[30] Gardet A, Benita Y, Li C, Sands BE, Ballester I, Stevens C, Korzenik JR, Rioux JD, Daly MJ, Xavier RJ (2010). LRRK2 is involved in the IFN-gamma response and host response to pathogens. J Immunol.

[31] Moehle MS, Webber PJ, Tse T, Sukar N, Standaert DG, DeSilva TM, Cowell RM, West AB (2012). LRRK2 inhibition attenuates microglial inflammatory responses. J Neurosci.

[32] Dzamko N, Inesta-Vaquera F, Zhang J, Xie C, Cai H, Arthur S, Tan L, Choi H, Gray N, Cohen P (2012). The IkappaB kinase family phosphorylates the Parkinson's disease kinase LRRK2 at Ser935 and Ser910 during Toll-like receptor signaling. PLoS ONE.

[33] Greggio E, Civiero L, Bisaglia M, Bubacco L (2012). Parkinson's disease and immune system: is the culprit LRRKing in the periphery?. J Neuroinflammation.

