# Supplementary material for: GTP binding controls complex formation by the human ROCO protein MASL1
Source: FEBS J. 2013 Nov 28;281(1):261–74. doi: 10.1111/febs.12593 (PMC3995007; doi:10.1111/febs.12593)
Supplement: Supplementary file 1 — Fig. S1. Nucleotide binding regulates complex formation. Fig. S2. Representative fluorescence spectra of purified wild type and K422A MASL1 in the presence and absence of 6 m GdHCl using an excitation wavelength of 280 nm. Fig. S3. SEC analysis of wild type MASL1 in the presence of 200 µm GTP or GMppCp. Fig. S4. FACS analysis quantification. [file febs0281-0261-sd1.zip › febs12593-sup-0002-FigS1-S4.pdf]

the FEBS Journal
www.febsjournal.org

GTP binding controls complex formation by the human ROCO protein MASL1

Sybille Dihanich, Laura Civiero, Claudia Manzoni, Adamantios Mamais, Rina Bandopadhyay, Elisa Greggio and Patrick A. Lewis

DOI: 10.1111/febs.12593

Supplementary Figure 1

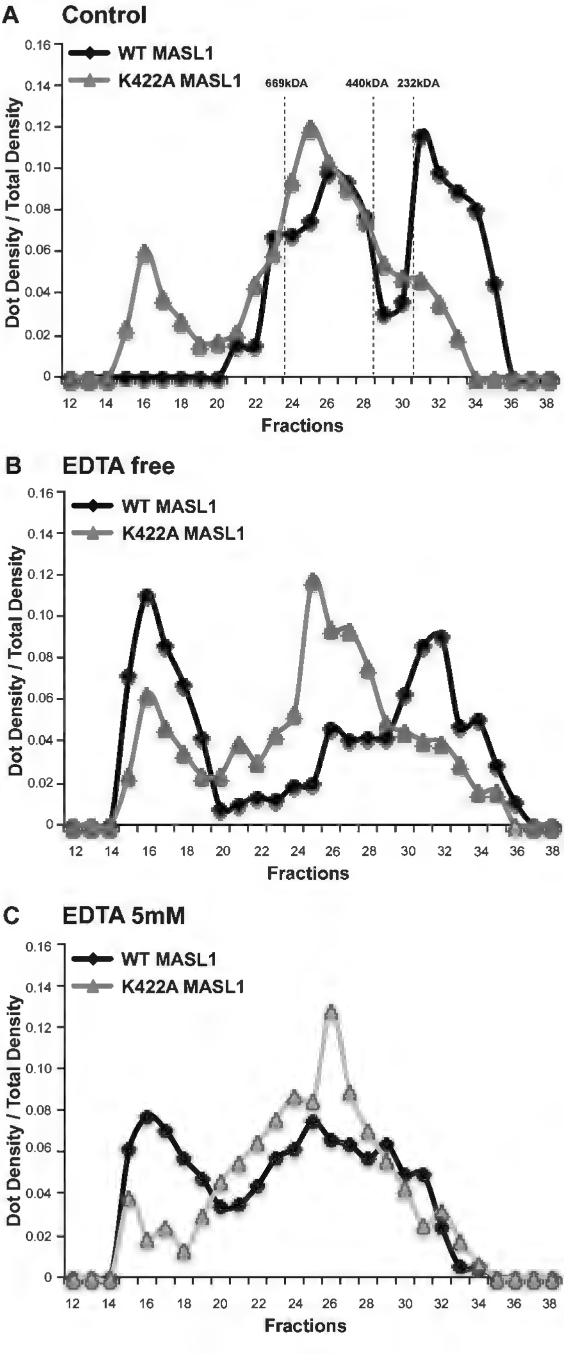

Supplementary Figure 2

A Wild type +/- GdHCl

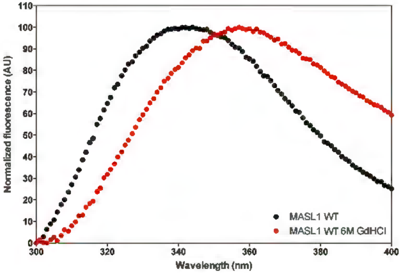

B K422A +/- GdHCl

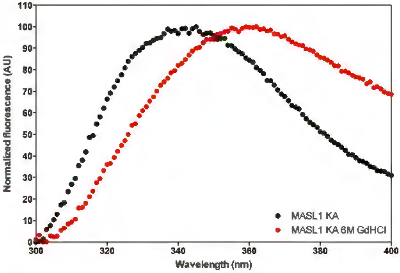

C Native Wild type and K422A

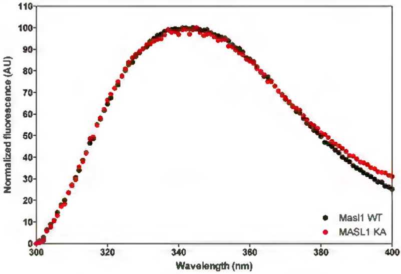

Supplementary Figure 3

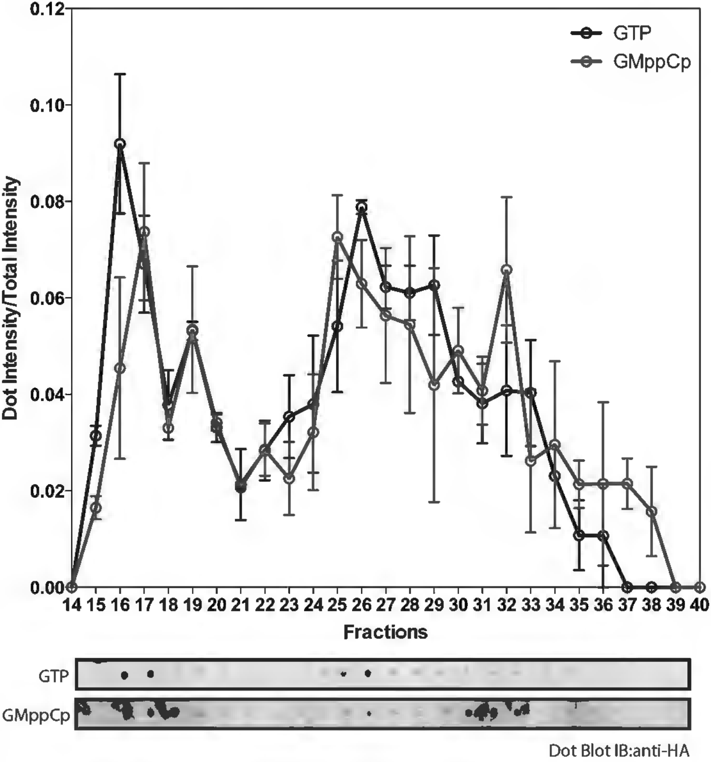

Supplementary Figure 4

A untransfected Control cells

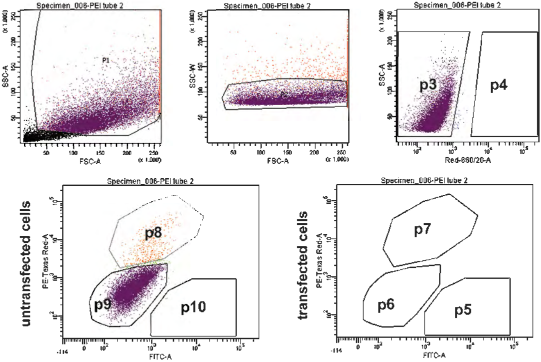

B MASL1 FACS analysis data

		WT MASL1			K422A MASL1			CONTROL		
		sample 1	sample 2	sample 3	sample 1	sample 2	sample 3	sample 1	sample 2	sample 3
p3	untransfected cells	138,000	150,860	80,815	244,319	128,703	199,107	15,635	13,907	13,762
p8	necrotic cells	4,425	3,862	3,129	6,545	4,981	5,510	1,070	443	594
p9	live cells	132,314	145,796	76,954	233,990	122,199	190,299	14,127	13,338	12,989
p10	apoptotic cells	1	8	2	3	4	6	3	2	0
p4	transfected cells	5,332	5,194	4,472	6,709	5,187	6,571	1	0	1
p7	necrotic cells	250	223	256	840	333	803	0	0	0
p6	live cells	4,966	4,875	4,141	5,648	4,746	5,559	1	0	0
p5	apoptotic cells	0	1	0	0	1	0	0	0	0
